# Hypoglycemic and hypolipidemic activities of aqueous extract of flowers from *Nycantus arbor-tristis* L. in male mice

**DOI:** 10.1186/s12906-015-0807-0

**Published:** 2015-08-19

**Authors:** Bramanage Sachini Rangika, Pavithra Dilakshini Dayananda, Dinithi Champika Peiris

**Affiliations:** Department of Zoology, Faculty of Applied Sciences, University of Sri Jayewardenepura, Gandoawilla, Nugegoda 10250 Sri Lanka

**Keywords:** Hypoglycemic, Hypolipidemic, Diabetes mellitus, *Nyctanthes arbor-tristis*, Aqueous extract of flower

## Abstract

**Background:**

Boiled aqueous extract of flowers (AEF) from *Nyctanthes arbor-tristis* L. are used in Sri Lankan traditional Ayruvedic Medicine to manage diabetes mellitus. AEF has widely been used as a folk medicine for the treatment of various ailments due to its therapeutic activity. However, little is known concerning therapeutic activity of the extract as well as its underline mechanisms and safety. Diabetes is known to increase low-density cholesterol and decrease high-density cholesterol thus triggering coronary diseases. Hence, the primary objective of the present study is to investigate the hypoglycemic and hypolipidemic activities of the AEF.

**Methods:**

AEF was prepared and male mice (*n* = 9 group) were gavaged either with 250, 500 and 750 mg/kg of AEF or distilled water (DW). Subsequently, fasting and random blood glucose concentrations were determined. To investigate mechanisms of actions of AEF, animals were orally administered with 500 mg/kg or the vehicle (DW) and glucose tolerance was performed before and after glucose challenge. For further studies, *in vitro* alpha-amylase assay and glucose absorption from the gastrointestinal tract were performed using 500 mg/kg of the extract. Additionally, glycogen content in the liver and skeletal muscles, a complete lipid profile assay, and toxicological and biochemical parameters were conducted after a chronic study.

**Results:**

Five hundred mg/kg and 750 mg/kg of AEF significantly (*p* < 0.01) reduced fasting blood glucose levels respectively by 49 and 39 % at 4 h post-treatment, while 500 mg/kg of AEF also decreased the random blood glucose level significantly (*p* <0.01) by 32 % at 4 h post-treatment. AEF significantly inhibited glucose absorption by 85 % from the intestine and increased diaphragm uptake of glucose by 64 %. The extract also exhibited inhibition (16.66 %) of alpha-amylase enzyme activity. It also decreased the level of total cholesterol (by 44.8 %), triglyceride (by 53 %) and increased (by 57 %) the high-density lipoprotein cholesterol. Treatment with AEF did not induce any overt signs of toxicity or hepatotoxicity.

**Conclusion:**

Results the present study indicated that AEF possess hypoglycemic and hypolipdemic properties. Therefore, AEF could be used as an alternative medicine in management of diabetes mellitus.

## Background

Diabetes mellitus is a major global burden with 382 million people suffering from the disease at the end of 2013 and is projected to increase up to 592 million by 2035 [1]. More than 80 % of the diabetes deaths occur in the low and middle-income countries and more than 471 billion US dollars are spent in the health care expenditure worldwide for diabetes [2]. Type 1 diabetes is prevalent among Northern European countries while Type 2 diabetes is prevalent in African and South Asian countries [3]. Sri Lanka, being a South Asian country, type 2 diabetes is prevalent. Type 2 diabetes is also known as non-insulin-dependent diabetes and is caused by the body’s ineffective use of insulin [4]. One in five adult in Sri Lanka is suffering either from diabetes or pre-diabetes [5]. The American Diabetes Association defines a pre-diabetic individual as an individual with blood glucose levels higher than normal (impaired fasting glucose between 100 and 125 mg/dl, impaired glucose tolerance between 140 and 199 mg/dl, and HbA1c between 5.7 and 6.4 %) but not high enough to be considered diabetic (impaired fasting glucose between >126 mg/dl, impaired glucose tolerance between >200 mg/dl, and HbA1c between >6.5 % [1]. Diabetes tends to increase low-density lipoprotein cholesterol and decrease high-density lipoprotein cholesterol levels in serum triggering coronary occlusions and blocks. Therefore, it is important to control not only blood glucose levels but also blood lipid levels. It has been shown that current treatment for diabetes with synthetic hypoglycemic agents can cause adverse effects including hypoglycemia, gastrointestinal disturbances, renal toxicity and hepatotoxicity [6]. Hence, search of plant formulations with minimum toxicity remains a challenge. Various plant species have been known to possess potent hypoglycemic activity. Therefore, plant remedies have been used as an alternative treatment for diabetes mellitus in Ayurvedic and traditional medicine by different cultures around the world [7].

*Nyctanthes arbor-tristis* (Family-*Oleaceae*), commonly known as night-flowering Jasmine or Sepalika (in Sinhala) distributed in tropical and sub-tropical regions of Sri Lanka. It is one of the well-known medicinal plants for its antidiabetic properties [8]. It is a large shrub growing into about 10 m tall with a flaky grey bark. Flowers are fragrant and have a 5–8 lobes, white corolla with an orange center [9, 10]. Different parts of *N. arbor*-*tristis* plants are used in Ayurveda, Siddha-Ayurveda and Unani systems of medicine as a digestives, antidote to reptile venoms, mild bitter tonic, laxative, diaphoretic, diuretic and arthritis [11]. The leaves extract has many proved pharmacological effects like anti-bacterial [10], analgesic, anti-inflammatory [11], anti-diabetic [8, 12], anti-arthritic [13], antioxidant [10], hepatoprotective [14] and antispasmodic activities [12]. Flower extract is also used as a stomachic, carminative, astringent to bowel, antibilious, expectorant, hair tonic and in the treatment of piles and various skin diseases [7]. Boiled aqueous extract of flowers are known to have sedative effects [12], while fresh flowers and dried leaves of *N. arbor-tristis* extract demonstrated antispasmodic activity, antihelmintic activity, while stem bark extracts exhibit anti-diabetic activity and anti-inflammatory activity [9].

It has been shown that chloroform extracted *N. arbor-tristis* flowers reduce blood glucose level in diabetic induced rats [14]. In traditional Sri Lankan Ayurveda medicine; boiled aqueous extract of *N. arbor-tristis* flowers had been used in the management of type 2 diabetes mellitus [14]. But thus far, this is not scientifically proven and the mechanisms by which *N. arbor-tristis* can reduce diabetes have not been established. Analyzing its antidiabetic and antilipidemic potentials could generate baseline data for development of a new therapy with minimum toxicity and high efficacy. Thus, the present study was conducted to investigate hypoglycemic and hypolipidemic roles of AEF from *N. arbor-tristis* in male mice. Further, we extended the investigation to study underling toxic effects of AEF.

## Methods

### Plant material

Fresh *N. arbor-tristis* flowers were collected early morning (6.00–7.00 a.m.) from home gardens of Bentota (6.4200° N, 80.0000° E), Sri Lanka between February and April 2013 and was identified and authenticated by Prof. B.M.P Singhakumara, Dept. of Forestry and Environment Science, University of Sri Jayewardenepura. A voucher specimen (SP no: DP/01) was deposited in the Department of Zoology, University of Sri Jayewardenepura.

### Method of extraction

Collected flowers were thoroughly washed with water and dried in shade at room temperature. Dried flower materials were milled to fine powder using a mechanical grinder and 2 g of powder was infused in 100 ml of boiling water for 30 min. Brown colour AEF was obtained and filtered using a muslin cloth (yield 21.42 % W/V).

### Animals

Healthy, adult ICR (Imprinting Control Region) male mice (weight 30–40 g) were used for all experiments. They were maintained under standard animal house conditions (temperature: 26–30 °C, photoperiod: approximately 12 h natural light per day and relative humidity: 55–60 %) with continuous access to pelleted food (Vet House Ltd, Colombo, Sri Lanka) and tap water. Research was conducted in accordance with the internationally accepted principles for care and use of laboratory animals and guidelines. Ethical approval (ethical clearance no.711/13) was obtained by the institutional ethical review committee (Ethical Review Comittee, Faculty of Medicine, University of Sri Jayewardenepura).

### Dosages & treatment

Doses of 250 mg/kg, 500 mg/kg and 750 mg/kg of AEF were prepared directly from the hot infusion. The mid dose or 500 mg/kg of AEF was selected based on dose recommended by traditional Ayruvedic physicians of Sri Lanka in prescribing herbal decoctions. To evaluate fasting blood glucose level, 36 male mice were randomly divided into 4 groups (*n* = 9/group) and treated either with 1 ml of DW or 250 mg/kg, 500 mg/kg and 750 mg/kg of AEF. Since the maximum glucose reduction was observed with animals treated with 500 mg/kg, this dose was used for further experiments. Hence, only 18 mice were used for each experiment with 9 mice per group.

### Effects of AEF on fasting & random blood glucose levels

After an overnight fast for 16 h with free access to water mice were treated either with 3 doses of AEF or DW. Using aseptic conditions, blood samples were collected from animals to determine blood glucose levels at 1 h prior to the treatment and 2 h, 4 h post-treatment. Average blood glucose value was taken after triplicate measurement [15]. To evaluate the random blood glucose level, animals were gavaged only with 500 mg/kg AEF and DW and same procedure was used without fasting the animals [16].

### Effects of AEF on glucose tolerance

Method previously described by Okaine et al. [17] was used. After an overnight fast of mice for 16 h, animals were assigned randomly into 2 groups (*n* = 9). They were orally administerd either with DW or 500 mg/kg of AEF and 30 min after gavaging, all mice were loaded with 2 g/kg glucose solution. Blood samples were collected and glucose levels 1 h prior to treatment and 2 h, 4 h after glucose challenge were determined.

### Effects of AEF on gastrointestinal glucose absorption

Two groups of mice were orally administrated either with 500 mg/kg of AEF or DW and after 30 min, mice were treated orally with 10 ml/Kg of 50 % glucose. Two hours following the treatment, mice were sacrificed and glucose absorption by the intestine was determined [14].

### Effects of the AEF on liver & skeletal muscle glycogen content

Two sets of mice (*n* = 9) were treated orally either with 1 ml of DW or 1 ml of 500 mg/Kg AEF for 30 consecutive days. All the mice were sacrificed and portions of their livers and gastrocnemius muscles were removed for glycogen analysis [18].

### Effects of AEF on diaphragm uptake

Eighteen mice were randomly divided in to 2 groups (*n* = 9) and were treated either with the plant extract or DW for 30 days. Animals were sacrificed on day 31 and isolated diaphragms incubated in 1 g/l glucose solution to assess glucose uptake [19]. Glucose concentrations was measured immediately prior to incubation and 30 min following incubation.

### Effects of the extract on α-Amylase assay *in vitro*

The α-amylase inhibition assay for the AEF was performed according to the methods described by Rahimzadeh et al. [20] with slight modifications. Briefly, the assay mixture consisted of 500 μl of 1 % starch solution, 400 μl of 0.1 M sodium phosphate buffer (pH 7.0), 50 μl of plant extract dissolved in dimethyl sulfoxide (DMSO) and 50 μl of pancreatic α-amylase (Sigma, St. Lous, USA) solutions (2 U/ml). Then, the reaction medium was incubated at 37 °C for 10 min followed by addition of 3 ml of 3,5- dinitrosalicylic acid (DNS) color reagent. Finally, the solution was placed in a boiling water bath for 5 min, diluted with 20 ml of distilled water and the absorbance was measured at 540 nm. Absorbance of a control sample was prepared accordingly without plant extract and acted as negative control. The standard antihyperglycemic agent acarbose was used as positive control. The experimental extract and acarbose were tested with varying concentrations from 1.25 to 5 mg/ml. The results were expressed as percentage inhibition using the following formula:

$$ \mathrm{Inhibition}\kern0.5em \left(\%\right)=\frac{\left[\mathrm{A}\mathrm{c}-\mathrm{A}\mathrm{s}\right]\kern0.5em \mathrm{X}\kern0.5em 100}{\mathrm{Ac}} $$

where Ac is the absorbance of the control reaction without sample and As is the absorbance of the sample. IC_50_ value, defined as the sample concentration (mg/ml) at which 50 % inhibition of the enzyme activity occurs, was calculated from the graph plotting enzyme inhibition against sample concentration. All tests were carried out for three sample replications and the results were averaged.

### Effects on serum lipid profile

Blood samples from 2 groups of mice (*n* = 9) were centrifuged at 4000 rpm for 20 min to separate serum to determine total cholesterol (TCH), high-density lipoprotein cholesterol (HDL-CH), low-density lipoprotein cholesterol (LDL-CH), and triglycerides (TG) using UV spectrophotometer (Labomed, INC, Los Angeles, USA) method [21].

### Toxicological and biochemical parameters

Two groups of mice were orally treated (between 9.00 a.m. and 10.00 a.m.) either with 1 ml of 500 mg/kg of AEF or 1 ml of DW for 30 consecutive days. Animals were observed daily between 11.00 a.m. and 12 noon for any overt sign of toxicity (diarrhoea, salivation, lachrymation, tremors, ataxia, loss of fur, change of fur colour, postural abnormalities or behavioural changes), stress (fur erection and exophthalmia), aversive behaviours, during the treatment period. Percentage of body weight gain, and food and water intake was determined during the study period.

Upon autopsy of animals, blood was collected from heart puncture to tubes containing heparin. Plasma was separated and alanine aminotransferase (ALT) and aspartate aminotransferase (AST) concentration were determined [22]. Subsequently, liver, kidney, spleen, heart and testes were removed from each mouse. Organs were blotted free of blood and wet weights were recorded. The organ weights were expressed as percentage of the organ weight per 1 g of body weight. Parts of liver and kidney were fixed in Bouin’s fixatives for histopathological analysis. After routine processing, paraffin sections of 4 μm thick sections were cut and stained with haematoxylin and eosin for microscopic examination.

### Statistical analysis

Statistical analysis was carried out using parametric one-way analysis of variance (ANOVA) followed by Mann–Whitney *U*-test using the statistical package, Minitab 14 for windows. The data were expressed as mean ± Standard Error Mean (SEM). The *p* value was set to ≤ 0.05.

## Results

### Effects on fasting & random blood glucose levels

**Fig. 1:**
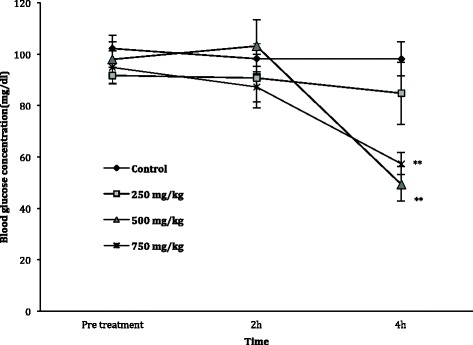
Effects of aqueous extreact of *N. arbor-tristis* flowers or control (DW) on fasting blood glucose levels of normal mice. Mice treated either with AFE of *N. arbor-tristis* at 250 mg/kg (□), 500 mg/kg (∆), 750 mg/kg (**□**) or DW (–). Glucose concentration (mg/dl) were measured prior to treatment and 1 h, 4 h post-treatment. Results are expressed as mean ± SEM; **P < 0.01

**Fig. 2:**
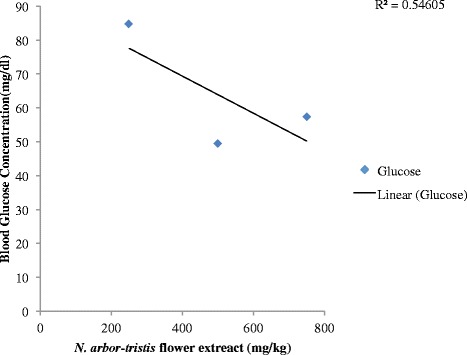
Linear regression analysis of *N. arbor-tristis* or distilled water on fasting blood glucose levels of mice. Mice were treated once either with 250 mg/kg, 500 mg/kg and 700 mg/kg of AFE or DW. Fasting blood glucose level was measured prior to treatment and 2 h, 4 h post-treatment. Results are expressed as mean ± SEM. (R^2^ = 0.546)

Mice treated with 250 mg/kg of AEF had no effect on blood glucose level during the experimental time period. But, 500 and 750 mg/kg of plant extract showed significant (*p* < 0.01) blood glucose decreasing efficacy at 4 h post-treatment. The blood glucose levels were reduced respectively by 49 and 39 % when compared to control. The AEF failed to inhibit fasting blood glucose level at 1 h or 3 h and hence a delayed effect is evident (Fig. 1). Figure 2 shows the dose dependent fasting blood glucose activity of AEF and linear regression analysis showed that the effect was not dose-dependent (R^2^ = 0.546).

**Fig. 3:**
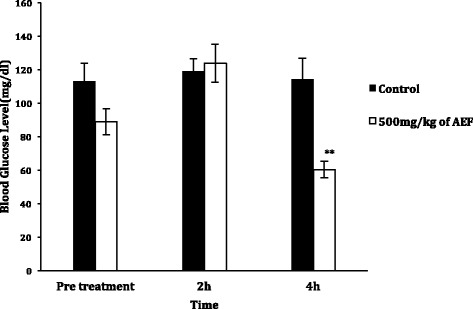
Effects of *N. arbor-tristis* or DW on random blood glucose levels of mice. Mice were treated once either with 1 ml of 500 mg/kg of AFE or distilled water. Random glucose level was measured prior to treatment and 2 h, 4 h post-treatment. Results are expressed as mean ± SEM. **P < 0.01

Treatment with effective dose (500 mg/kg) of AEF of *N. arbor-tristis* significantly (*p* < 0.01) reduced the random blood glucose level of mice by 32 % at 4 h thus indicating a delayed glucose-inhibiting efficacy (Fig. 3).

### Effects of AEF on glucose tolerance test

As shown in Table 1, the initial fasting blood glucose levels of both groups did not show any apparent difference. Similarly, treatment of mice with 500 mg/kg of AEF did not alter the fasting blood glucose level at 2 and 4 h following oral glucose challenge compared to control.

**Table 1:** Effect of *N. arbor-tristis* aqueous extract of flower on oral glucose tolerance test in mice. Glucose concentration (mg/dl)

Treatment	Pre-treatment	Post treatment		
		1 h	3 h	5 h
Control	113.5 ± 14.4	168.2 ± 24.3	87.13 ± 1.91	82.11 ± 3.77
AEF	86.87 ± 6.75	155.5 ± 13.9	91.03 ± 6.90	138.4 ± 12.2

Values are expressed as mean ± SEM, *n* = 9. Control groups received distilled water and treatment group received 500 mg/kg of the extract. No significant difference was observed between the groups. The data was analyzed by parametric method-ANOVA

### Effect of AEF on diaphragm and gastrointestinal glucose uptake

AEF produced a significant (*p* < 0.05) increase in diaphragm uptake of glucose compared to control. Similarly, AEF significantly (*p* < 0.05) inhibited glucose absorption by 83 % from the intestinal lumen 2 hours following treatment compared to control (Table 2).

**Table 2:** Effect of aqueous flower extract from *N. arbor-tristis*on gastrointestinal and diaphragm glucose uptake, liver and skeletal muscle glycogen content in mice

Parameters	Control	Treatment
Gastrointestinal absorption of glucose (mg/dl)	18.95 ± 2.85	132.3 ± 16.1*
Diaphragm uptake of glucose (mg/dl)	204.5 ± 66.1	578 ± 83.4*
Glycogen content in the liver (mg/dl)	2.238 ± 0.247	2.643 ± 0.284
Glycogen content in skeletal muscle (mg/dl)	0.523 ± 0.091	0.419 ± 0.076

The data are given as mean ± S.EM (*n* = 9). Values are statistically significant at *p < 0.05. Control group was given distilled water while test group was given 500 mg/kg of the extract. To measure diaphragm glucose uptake, and glycogen content in liver and skeletal muscles, mice were treated for 30 days. For measurement of intestinal glucose absorption, mice were treated with acute doses. The data was analyzed by parametric method-ANOVA

### Effects of AEF on liver and skeletal muscle glycogen content

Within group analysis revealed that AEF treated animals did not show significant alteration in liver or skeletal muscle glycogen content when compared to their respective controls. The results are depicted in Table 2.

### Effects of AFE on *in vitro* α-Amylase assay

AFE produced a dose response inhibition of *in vitro* α-amylase activities. Alpha-amylase activity was significantly inhibited by 16.67 and 33.33 % respectively at 500 mg/kg (*p <* 0.05) and 750 mg/kg (*p* < 0.01) AEF (Table 3).

**Table 3:** *In vitro* α-amylase inhibitory effect of aqueous flower extract from *N. arbor-tristis*

Concentration mg/kg	α-amylase inhibitory activity (%)
250 mg/kg	8.33
500 mg/kg	16.67*
750 mg/kg	33.33^**^

Values are statistically significant at *p < 0.05, ^**^ *p*<0.01. Control group was treated with distilled water while test groups were treated with 250, 500 & 750 mg/kg of the extract. To measure diaphragm glucose uptake, and glycogen content in liver and skeletal muscles, mice were treated for 30 days. For measurement of intestinal glucose absorption, mice were treated with acute doses. The data was analyzed by parametric method-ANOVA

### Effects of AEF on serum lipid profile

Table 4 shows the serum levels of TCH, TG, LDL-CH and HDL-CH in normal and experimental animals of each group. A significant (*p* < 0.01) reduction in TCH and TG (by 44.88 and 53 % respectively) were evident in the treated group when compared to the control mice. Similarly, AEF significantly (*p* < 0.05) increased serum HDL-CH level by 57.27 % but did not exert any effect on serum LDL-CH levels.

**Table 4:** Effects of aqueous flower extract from *N. arbor-tristis* on lipid profile parameters, biochemical parameters and organ weights of mice after 30 days of treatment

Parameters	Control	Treatment
Lipid profile parameters		
Total Cholesterol (mg/dl)	127.9 ± 16.5	100.8 ± 11.5**
Triglycerides (mg/dl)	79.46 ± 7.46	37.32 ± 7.85*
HDL-CH (mg/dl)	43.073 ± 22.7	100.8 ± 25.2*
LDL-CH (mg/dl	29.73 ± 5.84	27.3 ± 10.8
Biochemical parameters		
ALT (IU/l)	2.968 ± 0.654	2.476 ± 0.42
AST (IU/l)	12.618 ± 0.438	12.569 ± 0.325
Organ weights		
Liver (g)	2.2343 ± 0.0993	2.1950 ± 0.0687
Spleen (g)	2.2288 ± 0.0177	2.2538 ± 0.0207
Kidney (g)	0.7100 ± 0.0316	0.2350 ± 0.0325
Testes (g)	0.2571 ± 0.0194	0.6475 ± 0.0177

The data are given as mean ± S.EM (*n* = 9). Values are statistically significant at *p < 0.05, **p < 0.01. Control group was given distilled water while treated group was given 500 mg/kg of the extract. AEF: aqueous extract of flowers. HDL-CH: High density lipoprotein cholesterol, LDL-CH – Low density lipoprotein cholesterol, ALT: Alanin aminotransferase levels; AST: serum Aspartate aminotransferase. The data was analyzed by parametric method-ANOVA

### Effects of AEF on biochemical and toxicological parameters

Oral ingestion of the extract did not alter serum ALT (control: 2.968 ± 0.654 versus treatment: 2.476 ± 0.420 IU/L) or AST (control: 12.618 ± 0.438 versus treatment: 12.569 ± 0.325 IU/L) levels (Table 4). Mice treated for 30 days with 500 mg/kg AEF did not show any overt signs of clinical toxicity, stress and aversive behaviours during the treatment period. Further, no deaths were observed. There was no suppression of body weight, water or food intake (Table 5) and organ weighst (Table 4) observed during the treatment period. Histopathological analysis of the liver and kidney portions after treatment with AEF did not exhibit any obvious effect when compared to control.

**Table 5:** Effect of aqueous extract of flowers from *N. arbor-tristis* on food intake, water intake and bodyweight change in mice after 30 days of treatment

		# of Weeks			
Parameters	Treatment	1	2	3	4
Food intake (g)	Control	0.1597 ± 0.0105	0.1556 ± 0.005	0.1543 ± 0.008	0.1553 ± 0.007
	Treatment	0.1757 ± 0.0180	0.1690 ± 0.0140	0.1653 ± 0.007	0.1657 ± 0.0135
Water intake (ml)	Control	0.2394 ± 0.0198	0.2369 ± 0.0176	0.2350 ± 0.0153	0.1753 ± 0.0776
	Treatment	0.2668 ± 0.0173	0.2574 ± 0.0235	0.2593 ± 0.0233	0.2516 ± 0.0249
Body weight (g)	Control	42.00 ± 1.25	42.00 ± 1.27	41.88 ± 1.29	41.63 ± 1.41
	Treatment	39.67 ± 1.72	39.44 ± 1.69	39.67 ± 1.65	40.22 ± 1.69

The data are given as mean ± S.EM (*n* = 9). No statistically significant differences were observed between values. Control group was given distilled water while treated group was given 500 mg/kg of the extract. The data was analyzed by parametric method-ANOVA

## Discussion

Morbidity and mortality due to diabetes is increasing worldwide and is the third cause of death after cancer [23]. Remedies available today for management of diabetes can lead to potential adverse effects. Therefore, plant derived components devoid of adverse effects have attracted particular attention as an alternative source to battle against diabetes. Thus, in lieu of searching of natural therapy, hypoglyceamic and hypolipidemic effects of AEF of *N. arbor-tristis* have been evaluated in the present study.

This study revealed that AEF of *N. arbor-tristis* possesses both hypoglycemic and hypolipidemic activities. In the current experiment, 500 mg/kg dose was equivalent to dosage prescribed by traditional Ayruvedic doctors in Sri Lanka and therefore, this dose was used as the mid dose to report hypoglycemic activity of the extract.

The results indicate that AEF produced significant reduction of blood glucose levels 4 h after oral administration in mice kept fasting overnight at doses of 750 and 500 mg/kg. Typically, traditional doctors advice patients to partake decoctions early in the morning and findings of this study align with their practice. It was observed that AEF exerted its hypoglycemic effects through none dose-dependant manner with the mid dose producing pronounced hypoglycaemic effect. Further, the extract did produce a delayed hypoglycemic effect after 4 h and such delayed activity had been reported previously in plants such as *Kokoona* zeylanica L. [24], *Strychnos henningsii* L. [25] and *Phylanthus debilis* L. [26]. High dose of the extract exhibiting less hypoglycemic activity than the mid dose may be due to receptor down regulation activity of the drug thus decreasing the number of receptors for a drug or presence of other phytochemical substances [27]. Further, it has been reported that presence of hypoglycemic and/or antagonistic substances in the extract may diminish hypoglyceamic effect [28]. Sireesha et al. [29] reported that higher doses of plant extract may posses various constituents that could result in non-specific increasing and reduction of blood glucose levels thus resulting in net effect of increasing blood glucose levels. But, future investigations are required to clarify delayed hypoglycemic effects produced by AEF.

Hypoglycemia may occur due to glucose oxidation via increase of metabolic actions as a result of high food consumption. Daily monitoring food intake of animals over one month period indicated that food intake was more or less similar in both control and treated groups. Hence, hypoglycemic activity observed during this study may not be due to low intake of carbohydrate diet [30]. Further, AEF did not increase the glucose tolerance after oral glucose challenge, which indicates that hypoglycemic activity exhibited by *N. arbor-tristis* may not to be due to insulin mimicacy action [31]. Previous qualitative phytochemical screening of *N. arbor-*tristis flowers exhibited the presence of abundant amount of manitol; a modified diterpenoid nycanthin, flavonoids, anthocyanins, nyctanthin, d-mannitol, tannin and glucose, carotenoid, glycosides viz 6β-hydroxylonganin and β-sitosterole posses hypoglycemic activity [32]. Therefore, hypoglycemic activity observed in the present study can be attributed to presence of flavonoids iridoide 6β-hydroxylonganin in *N. arbor-tristis* flowers [33].

The AEF had a gummy viscous appearance, which suggested presence of fiber [34]. *In vitro* studies had confirmed that glucose binds to dietary fibers even at very low glucose concentrations and these bound glucose are incapable of binding to specific transport protein for transportation. Thus, inhibiting glucose uptake from the gastrointestinal tract [35]. In addition, inhibition of intestinal glucose absorption may also be due to impaired intestinal Na^+^- glucose co-transporter as reported with synthetic phlorizin derivatives [36]. The estimation of glucose in mouse diaphragm is a commonly employed reliable method to study peripheral uptake of glucose. AEF also exhibited marked enhancement of glucose uptake by the diaphragm and was found to be more effective than insulin. It appears that AEF has direct peripheral action [37]. It has been also been suggested that direct glucose uptake by peripheral tissues is via receptor up regulation [38]. Therefore, increase glucose uptake by diaphragm can be attributed that long-term treatment of AEF that can lead to receptor up regulation.

AEF inhibited α- amylase activity in mice plasma *in vitro*. Inhibition of α- amylase activity can be attributed to several factors such as fiber concentration, presence of inhibitory constitutions and ecapsulation of starch and enzyme by fibers present in AEF [39]. Hence, hypoglycemic property of AEF can be linked with ability of the tannins and flavonoids reported in the flower extract to inhibit α-amylase enzyme. One possible mechanism of the extract to induce hypoglycemia could be via provoking inhibition of α- amylase activity [40]. This suggests that, it is possible for AEF to breakdown α linkage of polysaccharide to retard digestion and absorption of carbohydrates in the small intestine, thus reducing the increasing of blood glucose levels [35]. This hypothesis can further be supported by inhibition of glucose uptake observed in the gut with AEF treated animals. Thus, blood glucose reducing effects of AEF can be attributed to inhibition of intestinal glucose absorption or sensitivity of insulin in peripheral tissues or α- amylase inhibitory activity or combination of these mechanisms.

In diabetic condition hypertriglyceridemia and hypercholestermia are the common factors involved in the development of the atherosclerosis and coronary heart disease. In diabetes, increased levels of TCH are one of the major factors for coronary heart disease (hyperlipidaemia) and its incidence of atherosclerosis [41]. Interestingly, AEF reduced both serum TCH and TG levels and increased HDL-CH Level. The more prominent effect is the reduction in LDL-CH, which is a known triggering factor for coronary occlusion or its block. HDL-CH is protective cholesterol and is responsible for transportation of cholesterol [42]. Considering AEF’s effect on these lipid components, it can be assumed a potential hypolipidemic agent, which will be a great advantage both in diabetic condition as well as the associated atherosclerosis or hyperlipidemic conditions. Flavonoids from AEF [43] may augment activity of lecithin acyl transferase, which regulates blood lipids by incorporation of free cholesterol into HDL-CH thus increasing HDL-CH [41]. Similarly, saponin found in *N. arbor-tristis* [44] can find its way to cholesterol in intestinal lumen thus impairing cholesterol absorption from the intestine or bile acid causing reduction in extra hepatic circulation and increasing metabolism of cholesterol to sterol thorough their fecal excretion. Increase bile acid excretion offset by enhanced synthesis from cholesterol from the liver consequently lowers the plasma cholesterol [27].

Sub-chronic oral administration of mid dose of AEF did not show any overt sign of clinical toxicity, stress and aversive behaviours, hepatotoxicity (ALT and AST levels, liver weight and histopathology) or renootoxicity (kidney weight and histopathology). It has been established that liver is the main organ where toxic compounds are detoxified. Liver damage and it’s recovery is usually assessed by measuring serum ALT and AST levels. In the present no comparable changed in the treated mice were detected indicating no hepatotoxicity. These findings were further confirmed by the histopathological examination on the liver, which revealed to be normal. Further there was no change in body weight, food and water intake during the study period, thus indicating that boiled aqueous extract of *Nyctanthes* flowers are safe oral herbal drug.

Present study demonstrated that AEF could exert hypoglycemic and hypolipidemic activities with minimum toxicity. The mechanisms for the hypoglycemic action of *N. arbor-tristis* flowers could be via inhibiting α-amylase, inhibiting glucose diffusion by adsorbing and trapping glucose in its fiber matrix and by increasing glucose transport across the cell membranes. It may support the claims made on folkloric uses of AEF of *N. arbor-tristis* in the treatment of diabetes mellitus in traditional Sri Lankan Ayurveda medicine. This study can be further extended to revel hyperglycemic effects with diabetic mouse model.

## Conclusion

From this study, we can conclusively state that AEF of *N. arbor-tristis* possesses hypoglycemic and beneficial hypolipidemic properties. Finally, it can be considered that AFE is safe for oral consumption and elicits promising hypoglycemic activity in animal experiments. However the nature of the active principle(s) responsible for all these positive effects requires further investigation.

## Abbreviations

AEF: Aqueous extract of flowers
DW: Distilled water
TCH: Total cholesterol
HDL-CH: High-density lipoprotein cholesterol
LDL-CH: Low-density lipoprotein cholesterol
TG: Triglycerides
ALT: Alanine aminotransferase
AST: Aspartate aminotransferase
